# Reliability of Urinary Excretion Rate Adjustment in Measurements of Hippuric Acid in Urine

**DOI:** 10.3390/ijerph110707036

**Published:** 2014-07-11

**Authors:** Annamaria Nicolli, Federica Chiara, Alberto Gambalunga, Mariella Carrieri, Giovanni Battista Bartolucci, Andrea Trevisan

**Affiliations:** Department of Cardiologic, Thoracic and Vascular Sciences, University of Padova, I-35128 Padova, Italy; E-Mails: annamaria.nicolli@unipd.it (A.N.); federica.chiara@unipd.it (F.C.); alberto.gambalunga@unipd.it (A.G.); mariella.carrieri@unipd.it (M.C.); giovannibattista.bartolucci@unipd.it (G.B.B.)

**Keywords:** hippuric acid, specific gravity, creatinine, urinary excretion rate, urinary volume, urinary flow rate

## Abstract

The urinary excretion rate is calculated based on short-term, defined time sample collections with a known sample mass, and this measurement can be used to remove the variability in urine concentrations due to urine dilution. Adjustment to the urinary excretion rate of hippuric acid was evaluated in 31 healthy volunteers (14 males and 17 females). Urine was collected as short-term or spot samples and tested for specific gravity, creatinine and hippuric acid. Hippuric acid values were unadjusted or adjusted to measurements of specific gravity, creatinine or urinary excretion rate. Hippuric acid levels were partially independent of urinary volume and urinary flow rate, in contrast to specific gravity and creatinine, which were both highly dependent on the hippuric acid level. Accordingly, hippuric acid was independent on urinary specific gravity and creatinine excretion. Unadjusted and adjusted values for specific gravity or creatinine were generally closely correlated, especially in spot samples. Values adjusted to the urinary excretion rate appeared well correlated to those unadjusted and adjusted to specific gravity or creatinine values. Thus, adjustment of crude hippuric acid values to the urinary excretion rate is a valid procedure but is difficult to apply in the field of occupational medicine and does not improve the information derived from values determined in spot urine samples, either unadjusted or adjusted to specific gravity and creatinine.

## 1. Introduction

Several methods are used to adjust urinary indices in spot samples according to the concentration/dilution of urine. To avoid variations in the urinary concentration due to altered water contents, the urinary excretion rate (UER) was recently proposed as a method [1] and shown to be suitable for specific age and demographic categories [2]. The UER is uses short-term, defined time sample collections and is calculated by multiplying the analyte concentration in the urine by the volume of the void after bladder emptying and then dividing by the duration of time the void accumulated in the bladder, assuming the bladder was completely emptied after each urination and that the entire sampling void volume is known [2]. This method is based on the mass sample and removes the variability due to the concentration/dilution of urine, particularly for analytes that are affected by urinary flow [3].

In clinical practice, the urine concentration of a solute is evaluated as a unit of volume (24-hour collection). On the other hand, several analytes measured in occupational medicine are excreted immediately after exposure, indicating that 24-hour collections could lead to underestimation of the results [4,5].

The collection of spot urine samples is the most common procedure used to assess chemical exposure at work. However, these samples are influenced by concentration/dilution due to factors such as renal physiology, the intake of foods and liquids, and perspiration.

Among the methods used to adjust spot samples due to concentration/dilution, specific gravity (SG) and osmolality are influenced by significant amounts of sugar or proteins and may lead to highly erroneous results [6]. At present, the most common procedure is adjustment to the level of creatinine.

The aim of the present study was to compare the adjustment of urinary levels of hippuric acid (HA), which is the main metabolite of toluene but can also be measured in non-exposed subjects because it is a final metabolite of several substances, to SG and creatinine with the adjustment to UER. Until recently, the UER has generally been applied to pesticides [1] and was shown to demonstrate good efficacy for monitoring exposure in children. The usefulness of UER in the field of occupational medicine, when applied to a large employed biological index of exposure such as HA, could help to avoid the reliability of concentration/dilution adjustment.

## 2. Materials and Methods

Thirty-one healthy volunteers (14 males and 17 females, 30.4 ± 2.9 years old) were recruited among PhD students and Occupational Medicine residents of the Department of Cardiologic, Thoracic and Vascular Sciences of Padua University. Volunteers were informed of the aim of the study, and informed consent was obtained. The subjects were asked to empty their bladders (a portion of this urine was collected and represented the spot sample), and accurate calculations were performed to assess the time and the volume between emptying of the bladder and the following micturition (short-term sample). The UER was also calculated for this sample. All samples were collected during the morning. Portions of the spot and short-term samples were stored at −20 °C until the collection of all samples was completed.

SG was determined using an AT315 URICON^®^ refractometer (Tokio, Japan), and creatinine was measured by means of a Perkin-Elmer lambda 5 model spectrophotometer (Boston, MA, USA) and a commercial kit (Boehringer, Mannheim, Germany) based on Jaffè’s basic picrate method. HA was analyzed by means of high-pressure liquid chromatography (HPLC) [7], using a Kontron Instruments (Zurigo, Switzerland) HPLC equipped with a UV Kontron HPLC Detector 430. Body mass index (BMI) was also determined for all subjects.

Urinary HA in short-term and spot samples was unadjusted (HA_un_) or adjusted to SG (HA_SG_) according to Elkins formula [6] and in relation to a SG standard of 1024 or creatinine (mg mmol creatinine^−1^, HA_cn_). Values in short-term samples were also adjusted to UER (HA_UER_), according to the Rigas formula [1].

Statistical analyses were carried out using Statsdirect 2.7.7 version software (Statsdirect Ltd, Altrincham, UK). The statistical evaluation was descriptive, if appropriate, or was performed using linear regression analysis (two sided). The 95% confidence interval (Fisher’s z transformation for r) was also determined. In all tests, a p value less than 0.05 was considered statistically significant.

## 3. Results

The age and BMI of the subjects, the concentration/dilution of urine in short-term and spot samples (defined by SG and creatinine) and the HA concentration (either adjusted or not) are reported in Table 1. Both types of samples showed, on the average, similar urine and HA concentrations.

**Table 1:** Characteristics of the subjects and their urine and HA excretion in short-term and spot samples.

	Mean ± SD		Range	
Age (years)	30.4 ± 2.9		25–36	
BMI	21.5 ± 3.0		17.7–29	
	short-term samples		spot samples	
	mean ± SD	range	mean ± SD	range
SG	1018 ± 9	1003–1032	1020 ± 7	1004–1033
creatinine mmol l^−1^	12.6 ± 8.9	1.2–30.2	14.0 ± 7.6	1.4–31.8
HA_un_ mg l^−1^	390.8 ± 427.4	9–1683	505.9 ± 606.3	25–2146
HA_SG_* mg l^−1^	533.3 ± 644.3	58–3253	612.1 ± 858.4	67–3737
HA_cn_ mg mmol^−1^	49.1 ± 85.7	3–409	38.5 ± 51.2	4–221
HA_UER_ mg	332.7 ± 443.7	28–2379		

*adjusted to a SG standard of 1024.

In short-term samples, the SG and creatinine levels were significantly inversely correlated with the urinary volume (*r* = −0.701, *p* < 0.0001, C.I. 95% −0.845 and −0.461 and *r* = −0.688, *p* < 0.0001, C.I. 95% −0.838 and −0.441, respectively) and UFR (*r* = −0.817, *p* < 0.0001, C.I. 95% −0.908 and −0.651, and *r* = −0.727, *p* < 0.0001, C.I. 95% −0.860 and −0.502, respectively) but not with the time of collection. Furthermore, contrary to what was observed for adjustments to SG, creatinine or UER, HA_un_ was significantly inversely correlated with urinary volume (*r* = −0.496, *p* = 0.0045, C.I. 95% −0.724 and −0.172) and UFR (*r* = −0.405, *p* = 0.0238, C.I.95% −0.664 and −0.059) but not with the time of collection. In addition, HA_un_ was slightly, but significantly, correlated to creatinine (*r* = 0.435, *p* = 0.0145, C.I. 95% 0.095–0.684) and SG (*r* = 0.396, *p* = 0.0276, C.I. 95% 0.048–0.657) for short-term samples but not spot samples.

Table 2 part A shows the correlation among HA-adjusted or non-adjusted values for spot *vs.* short-term samples, and the strongest correlation was observed for HA-unadjusted values in both samples (*r* = 0.817, *p* < 0.0001, C.I. 95% 0.651–0.908).

As shown in Table 2 part B, the correlations among HA-unadjusted or -adjusted values were compared separately according to whether the metabolite was detected in short-term or spot samples. The correlations in spot samples appeared better than those in short-term samples, and the strongest correlation was observed between HA values adjusted to creatinine or SG (*r* = 0.985, *p* < 0.0001, C.I. 95% 0.968–0.993).

**Table 2:** Correlation between HA excretion in spot and short-term urine samples unadjusted or adjusted to SG or creatinine.

Part A	Equation	*r*	*p*	C.I. 95% *
HA_un_ spot *vs.* HA_un_ short time	*y* = 1.159*x* + 52.757	0.817	<0.0001	0.651–0.908
HA_SG_ spot *vs.* HA_SG_ short time	*y* = 1.060*x* + 46.596	0.796	<0.0001	0.615–0.897
HA_cn_ spot *vs.* HA_cn_ short time	*y* = 0.405*x* + 18.546	0.679	<0.0001	0.427–0.833
**Part B**	**Equation**	***r***	***p***	C.I. 95% *
HA_SG_ short time *vs.* HA_un_ short time	*y* = 1.208*x* + 60.964	0.802	<0.0001	0.625–0.900
HA_SG_ spot *vs.* HA_un_ spot	*y* = 1.319*x* − 55.205	0.932	<0.0001	0.862–0.967
HA_cn_ short time *vs.* HA_un_ short time	*y* = 0.098*x* + 10.790	0.489	=0.0052	0.163–0.719
HA_cn_ spot *vs.* HA_un_ spot	*y* = 0.077*x* − 0.737	0.918	<0.0001	0.835–0.960
HA_cn_ short time *vs.* HA_SG_ short time	*y* = 0.103*x* − 5.980	0.777	<0.0001	0.583–0.887
HA_cn_ spot *vs.* HA_SG_ spot	*y* = 0.059*x* + 2.513	0.985	<0.0001	0.968–0.993

*Fisher’s z transformation

**Figure 1:**
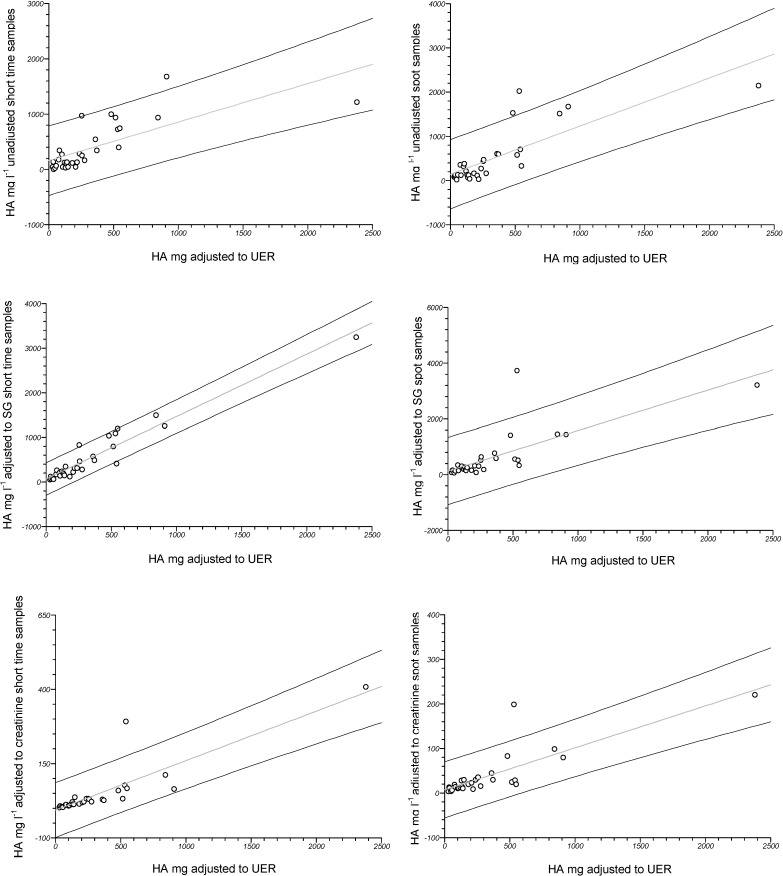
First line: correlation between HA_UER_ and HA_un_ in short-term (left: *y* = 0.697*x* + 158.937, *r* = 0.723, *p* < 0.0001) and spot samples (right: *y* = 1.086*x* + 144.414, *r* = 0.795, *p* < 0.0001). Second line: correlation between HA_UER_ and HA_SG_ in short-term (left: *y* = 1.399*x* + 67.645, *r* = 0.964, *p* < 0.0001) and spot samples (right: *y* = 1.452*x* + 128.947, *r* = 0.751, *p* < 0.0001). Third line: correlation between HA_UER_ and HA_cn_ in short-term (left: *y* = 0.167*x* − 6.306, *r* = 0.862, *p* < 0.0001) and spot samples (right: *y* = 0.094*x* + 7.099, r = 0.817, *p* < 0.0001). The 95% C.I. was calculated (Fisher’s z transformation) and shown for each correlation.

The correlation between HA_UER_ and HA, either adjusted or not, in both type of samples is illustrated in Figure 1. The strongest correlation for analytes measured in spot samples was observed after adjusting to creatinine (*r* = 0.817, *p* < 0.0001, C.I. 95% 0.651–0.908) and in short-term samples was observed after adjusting to SG (*r* = 0.964, *p* < 0.0001, CI 95% 0.925–0.982).

Finally, in data not shown, BMI was slightly correlated with SG (*r* = 0.360, *p* = 0.0469, C.I. 95% 0.006–0.633) and creatinine (*r* = 0.424, *p* = 0.0173, C.I.95% 0.082–0.677) in spot samples but not in short-term samples, and sex appeared slightly inversely correlated to creatinine in spot samples only (*r* = −0.380, *p* = 0.0348, C.I. 95% −0.647 and −0.030). Age was not found to influence concentration-dilution parameters (SG or creatinine) in either type of sample.

## 4. Discussion

HA is a urinary metabolite that is used as a common biomarker of toluene exposure; this compound is derived from benzoic acid metabolism as a result of the conjugation of the carboxylic group of benzoic acid with glycine [8,9,10,11,12,13,14]. The high urinary background level of HA due to the consumption of several foods [15], as well as environmental exposure to fuel vapors containing benzene, toluene, and xylene, is the reason why this metabolite was selected to study the reliability of UER adjustment without the need to recruit toluene occupationally exposed subjects.

Our results support the notion that adjustment to the UER is a valid method to avoid problems related to the concentration/dilution of urine for biological monitoring. Similar to traditional adjustment factors, adjustment to the UER takes into account the different behaviors of urinary metabolites (e.g., diffusion, active secretion, reabsorption).

The concentration, but not the mass, of an analyte excreted over time by active secretion is affected by urinary flow [3]. When passive diffusion plays a major role in renal output, the rate of excretion is directly proportional to the diuresis and the rate of fluid passage from the kidney to the urinary bladder. Contextually, the concentration of xenobiotics in the urine is generally less dependent on urine production, as the process of diffusion is determined by the equilibrium of partial pressure in the urine and plasma and the excretion of ionic compounds is strongly influenced by urinary pH variations, depending on the rate of urinary flow. Owing to a low urinary flow (<1 ml min^−1^), excretion can dramatically decrease when substances are eliminated by diffusion and reabsorbed, whereas secreted substances and those not able to be reabsorbed may not be appreciably affected. For substances in which the excretion rate is influenced by the UFR (*i.e.*, the concentration is less dependent on urinary flow), concentration adjustment with the SG may help to minimize the variability associated with urinary flow.

These results confirm those of other studies showing that creatinine and urinary solutes are not free from the effects of urinary volume [16] and are highly inversely correlated with UFR [17]. Furthermore, our results support the notion that urinary volume affects [18] the unadjusted urinary concentration of HA, but not if it is adjusted to SG or creatinine. In fact, not all urinary analytes show the same behavior [19] because they may be not independent of creatinine content and thus are not independent of urinary dilution. The results of the present research also confirm [17] that SG and creatinine are inversely correlated with urinary volume and the UFR, whereas HA is only weakly, although significantly, correlated to these measurements. Interestingly, HA appeared to be related to the urinary concentration in short-term collection samples but not spot samples. Moreover, we observed that SG and creatinine were dependent on urinary volume and the UFR, whereas HA was partially independent of these measurements for short-term collections and completely independent for spot samples, as demonstrated by the lack of any correlation between HA_SG_ and HA_cn_ with SG and creatinine.

In addition, a strong correlation was observed between adjusted and unadjusted HA values in short-term and spot samples, and the correlation between HA_un_ and HA_SG_ or HA_cn_ in short-term samples was quite different from that observed for spot samples. In fact, in spot samples, these correlations were very high (*r* > 0.9), with a close correspondence between HA_SG_ and HA_cn_, whereas in short-term samples, the correlations were weaker, particularly between HA_un_ and HA_cn_. The biological half-life of HA is approximately three hours [20]. During timed collections, rapid excretion is likely influenced by the time between collections and especially the volume, as demonstrated by the inversely correlation between HA_un_ and volume. This could be explained by the differences observed for correlations between HA adjusted or unadjusted values in spot samples compared to short-term samples.

Uptake of HA from the plasma occurs via the organic anion transporter 1 along the basolateral membrane of the proximal tubule; thereafter, it is actively secreted in the urine [21]. Assuming that urinary flow affects its concentration, but not its mass [3], the application of UER adjustment related to mass [1] should remove the variability in the urine. In fact, HA_UER_, as well as HA_SG_ and HA_cn_, did not correlate with urinary volume or UFR, in comparison to HA_un_. On the other hand, HA_UER_ was closely related to HA_un_, HA_SG_ and HA_cn_ in both collection types. This result shows that adjustment to the UER does not improve, regarding HA, the values determined in spot samples. Furthermore, this procedure is difficult to apply in the field of occupational medicine, especially because values unadjusted or adjusted to SG or creatinine may be utilized differently.

## 5. Conclusions

In conclusion, our data clearly show that the adjustment of HA, and likely all anionic organic compounds actively secreted by the tubule, to UER is reliable. On the other hand, it is difficult to apply this approach in the field of occupational medicine, even if HA_UER_ adjustment could be useful in a controlled trial. However, in agreement with previous reports [17,22,23], we believe that the use of spot samples adjusted to creatinine (or, in subjects not affected by tubular diseases, to SG) provides the best expression of crude values in occupational medicine, for evaluations of single individuals rather than a group. However, because creatinine levels vary according to age, sex, and race/ethnicity, as previously suggested [24], it is important that these values are compared with those of an appropriate reference group.
